# Identification of Immuno‐Diagnostic and Therapeutic Markers in Tumors and Inflammatory Diseases Based on Single‐Cell and Machine Learning Methods

**DOI:** 10.1155/humu/5519367

**Published:** 2026-10-21

**Authors:** Jinfen Tang, Denggao Yuan, Zhou Wu, Yangyang Xie, Jianfen Xu

**Affiliations:** ^1^ Department of Hematology and Oncology Ningbo No.2 Hospital Wenzhou Medical University Ningbo City Zhejiang Province China wmu.edu.cn; ^2^ Department of Hepatology Intervention Ningbo No.2 Hospital Wenzhou Medical University Ningbo City Zhejiang Province China wmu.edu.cn; ^3^ Department of Anus and Intestine Surgery Ningbo No.2 Hospital Wenzhou Medical University Ningbo City Zhejiang Province China wmu.edu.cn; ^4^ Ningbo Key Laboratory of Medical Research on Blinding Eye Diseases Ningbo Eye Institute Ningbo Eye Hospital Wenzhou Medical University Ningbo City Zhejiang Province China wmu.edu.cn

**Keywords:** colorectal cancer, immune microenvironment, in silico drug sensitivity, mitochondrial metabolism, prognostic signature, RiskScore

## Abstract

**Background:**

Chemotherapy is the mainstay of systemic treatment for colorectal cancer (CRC), but drug resistance and tumor heterogeneity markedly limit survival. Mitochondrial metabolism is increasingly recognized as a regulator of tumor progression, immune contexture, and therapeutic response. However, the prognostic relevance of mitochondrial metabolism–related transcriptional patterns and their relationships with chemotherapy‐response signatures and immune features in CRC remain incompletely understood.

**Methods:**

Clinical and transcriptomic data from The Cancer Genome Atlas colorectal cancer cohort (TCGA‐CRC) and Gene Expression Omnibus cohorts GSE17537 and GSE104645 were analyzed. A curated set of mitochondrial metabolism–related genes (MMRGs) were used to calculate an MMRG score, whereas genes upregulated in chemotherapy‐sensitive cases in GSE104645 were used to derive a drug‐sensitivity gene‐set score by single‐sample gene set enrichment analysis (ssGSEA). Weighted gene coexpression network analysis (WGCNA), differential expression analysis, univariate Cox regression, least absolute shrinkage and selection operator regression, and multivariate Cox regression were used to construct a five‐gene RiskScore. Prognostic associations were assessed using Kaplan–Meier curves, time‐dependent receiver operating characteristic curves, external validation, and subgroup analyses. Immune‐cell enrichment and pRRophetic‐predicted half‐maximal inhibitory concentration values were evaluated, and CD36 was examined using in vitro phenotypic assays.

**Results:**

Tumors showed lower MMRG scores than normal tissues, and higher MMRG scores were associated with better overall survival (OS). The drug‐sensitivity gene‐set score was higher in tumors but was not significantly associated with OS. A five‐gene signature (CD36, CPA3, FCRL2, POU2F2, and S100B) generated a RiskScore that separated OS outcomes in the TCGA training, testing, and overall cohorts, as well as GSE17537, although its time‐dependent discrimination varied across cohorts and follow‐up periods. Similar survival associations were observed within subgroups defined by stage, age, and sex; however, independent or incremental prognostic value was not evaluated. High‐risk tumors exhibited altered immune‐cell enrichment, and the RiskScore was correlated with pRRophetic‐predicted drug IC50 values after Benjamini–Hochberg correction. CD36 expression was higher in SW1116 than in NCM460 cells, and its knockdown inhibited proliferation, migration, and invasion. Mitochondrial function was not directly measured in these experiments.

**Conclusion:**

The five‐gene RiskScore was associated with OS, immune‐cell enrichment, and in silico drug‐sensitivity estimates in CRC. It may serve as a candidate tool for prognostic stratification, but its incremental clinical value and treatment‐predictive utility require prospective validation. The CD36 knockdown experiments support a tumor‐promoting phenotype in SW1116 cells but do not establish dependence on mitochondrial metabolic reprogramming.

## 1. Introduction

Colorectal cancer (CRC) remains one of the leading causes of cancer‐related mortality worldwide [1–3]. Though screening programs, endoscopic techniques, and perioperative management have been advanced significantly, patients are still detected at locally advanced or metastatic stages, and a substantial number of early‐stage patients eventually develop recurrence and distant metastasis [4, 5]. For these individuals, fluoropyrimidine‐based chemotherapy, often combined with oxaliplatin or irinotecan (e.g., FOLFOX and FOLFIRI), constitutes the cornerstone of systemic treatment and is increasingly complemented by targeted therapies such as anti‐VEGF or anti‐EGFR monoclonal antibodies, and in selected subsets, immune checkpoint inhibitors [6, 7]. These regimens have extended survival and improved disease control in a subset of patients; however, primary resistance and the rapid emergence of acquired resistance are frequently observed in clinical practice [8, 9]. Once chemoresistance develops, treatment options become limited, and patients often experience rapid disease progression and poor long‐term survival [10, 11]. Moreover, conventional clinicopathological parameters, including tumor–node–metastasis (TNM) stage and basic laboratory indices, are insufficient to accurately capture the biological heterogeneity of CRC or to reliably predict which patients will benefit from chemotherapy and which will relapse early [12, 13]. As a result, identifying robust, biologically grounded molecular signatures that can stratify CRC patients according to both prognosis and chemotherapeutic sensitivity has crucial significance, thereby enabling more refined risk classification and tailored treatment strategies.

Metabolic reprogramming underpins several cancer hallmarks, including adaptation to stress, metastasis, and therapy resistance [14, 15]. Although much attention historically focused on the “Warburg effect” and aerobic glycolysis, emerging evidence underscores that mitochondrial metabolism remains highly relevant in many cancer types—including CRC [16]. Mitochondria govern tricarboxylic acid (TCA) cycle, fatty acid oxidation, oxidative phosphorylation (OXPHOS), metabolite generation, and redox homeostasis, and they are intimately involved in the regulation of programmed cell death and cellular stress responses [17, 18]. In recent years, multiple studies have shown that specific mitochondrial metabolic states (e.g., elevated OXPHOS capacity or enhanced fatty acid oxidation) can confer resistance to cytotoxic drugs or targeted therapies by maintaining ATP production under stress, buffering reactive oxygen species (ROS), modulating mitochondrial outer membrane permeabilization, and reshaping apoptotic thresholds [19, 20]. In CRC, changes in mitochondrial biogenesis, mitochondrial DNA content, the expression of OXPHOS‐related genes, or dynamics of mitochondrial fusion/fission have been associated with more aggressive phenotypes, higher metastatic potential, and worse prognosis [21]. Yet despite these observations, the majority of existing prognostic or predictive models in CRC have focused on immune or stromal gene signatures, glycolysis/lipid‐metabolism gene sets, or epithelial–mesenchymal transition (EMT) markers, whereas systematic, mitochondria‐centric analyses—especially those linking mitochondrial metabolism with chemoresistance and tumor immunity—remain scarce [22, 23].

Recent CRC studies have developed mitochondrial‐related gene signatures for estimating prognosis, characterizing the immune microenvironment, and computationally predicting treatment response [24, 25]. Most of these models were derived primarily from tumor transcriptomes and were optimized for survival classification or immunotherapy‐related inference. Relatively few studies have combined a chemotherapy response–annotated dataset with mitochondrial gene‐set scoring, coexpression network analysis, external survival validation, and immune profiling. Accordingly, the present study was designed as a chemoresponse‐informed prognostic investigation rather than as a direct clinical predictor of chemotherapy benefit.

These insights together suggest that mitochondrial metabolism may act as a central integrator linking chemotherapeutic response, malignant progression, and immune‐microenvironment modulation in CRC. Mitochondrial activity influences intrinsic tumor‐cell behaviors (such as proliferation, apoptosis tolerance, motility, and EMT) and also shapes features of the tumor microenvironment (TME), such as nutrient availability, oxygen consumption, redox balance, and the availability of metabolites that affect immune‐cell function [26–28]. For example, mitochondria‐derived metabolites, ROS, and mitochondrial dynamics can modulate antigen‐presenting cell activity, T‐cell activation/exhaustion, myeloid‐cell polarization, and immune‐suppressive niche formation [29, 30]. Simultaneously, exposure of CRC cells to chemotherapy imposes metabolic and oxidative stress that may selectively favor cell subpopulations with more flexible or OXPHOS‐high mitochondrial phenotypes, facilitating the emergence of drug‐tolerant clones [31]. Hence, it is plausible that transcriptional programs reflecting mitochondrial metabolism—especially in relation to chemoresistance and immune contexture—could be harnessed to derive a gene signature associated with chemotherapy response–related transcriptional features, immune characteristics, and long‐term prognosis [32]. Although several mitochondrial signatures have been reported, coordinated evaluation of these features remains limited.

The present study is aimed at developing and validating a prognostic signature based on mitochondrial metabolism–related genes (MMRGs) associated with overall survival (OS) in CRC and to characterize its relationships with chemotherapy response–derived transcriptional features, immune‐cell enrichment, and in silico drug sensitivity. Clinical and RNA‐sequencing data from The Cancer Genome Atlas (TCGA) were integrated with GSE104645, which contains first‐line chemotherapy response annotations, and the independent survival cohort GSE17537. Prognostic performance was evaluated in internal, external, and clinical subgroup analyses. Because no direct comparison with a conventional clinicopathological model or prospective treatment‐response validation was performed, the analysis was intended to generate hypotheses for future validation rather than to establish clinical independence or guide treatment selection.

## 2. Materials and Methods

### 2.1. Acquisition of Data and Preprocessing

TCGA database was accessed to obtain RNA‐seq expression profiles and clinical data for colon adenocarcinoma (COAD) and rectal adenocarcinoma (READ). Samples without full survival time or status were deleted, retaining only those with survival time greater than 0 days. RNA‐seq data were converted to fragments per kilobase of transcript per million mapped reads and log2‐transformed. A total of 589 tumor samples and 51 normal tissue samples were included.

The GSE17537 microarray dataset was downloaded from the Gene Expression Omnibus (GEO) database and used exclusively for external OS validation. Probe identifiers were converted to gene symbols, expression values were summarized at the gene level, and samples without complete OS data were excluded, leaving 55 tumor samples. GSE104645 was used to identify chemotherapy response–associated genes and to derive the drug‐sensitivity gene‐set score. GSE104645 was not used to validate the concordance between pRRophetic‐predicted IC50 values and observed clinical responses.

A curated set of 1234 MMRGs from a previous study was used in this analysis [33].

### 2.2. Differential Gene Expression Analysis

Based on the transcriptomic data from the GSE104645 dataset, differential expression analysis was performed. First, samples were divided into two groups based on chemotherapy efficacy: The “Sensitive” group (*n* = 112) included those with partial response (PR) and complete response (CR), whereas the “Resistant” group (*n* = 69) included samples with stable disease (SD) and progressive disease (PD). Samples with missing or unassessed efficacy (not evaluated) were excluded from the analysis.

Differential expression analysis of GSE104645 was performed using the “limma” package [34]. Group status (sensitive vs. resistant) was included as the explanatory variable, and the contrast was specified as sensitive minus resistant. The empirical Bayes method was used to obtain moderated gene‐wise statistics. A positive log2 fold change indicated higher expression in the sensitive group, whereas a negative value indicated higher expression in the resistant group. Genes with *p* < 0.05 were considered chemotherapy response–associated differentially expressed genes (DEGs). For the TCGA tumor‐versus‐normal comparison, DEGs were identified using “limma” with thresholds of |log2 fold change| > log2 (1.5) and Benjamini–Hochberg − adjusted *p* < 0.05.

### 2.3. Single‐Sample Gene Set Enrichment Analysis (ssGSEA)

The ssGSEA scores for MMRGs in the TCGA‐CRC cohort were computed with the ssGSEA method from the GSVA package in R [35]. This method was used to derive an individual‐level MMRG score for each sample. Additionally, the upregulated genes in the sensitive group were used to construct a feature set, which was then applied to derive an individualized drug sensitivity score.

### 2.4. Weighted Gene Coexpression Network Analysis (WGCNA)

Applying the pickSoftThreshold function in the WGCNA package, gene modules related to the feature scores were identified by WGCNA and soft‐thresholding power (*β*) was estimated [36]. Based on this parameter, hierarchical clustering was carried out to construct gene modules, setting the minimum module size to 60 genes (minModuleSize = 60). Associations between module eigengenes and the two feature scores were subsequently quantified. The green module was retained because it showed significant joint associations with both the MMRG score and the drug‐sensitivity gene‐set score, rather than because it had the largest correlation with each individual trait.

### 2.5. Enrichment Analysis

The genes from the selected modules were intersected with DEGs from the TCGA dataset to obtain a set of candidate genes with common biological significance. Subsequently, Gene Ontology (GO) and Kyoto Encyclopedia of Genes and Genomes (KEGG) enrichment analyses were conducted using the “clusterProfiler” R package [37].

### 2.6. Risk Model Construction and Validation

OS‐associated genes were first screened using univariate Cox regression. Candidate genes were then refined using least absolute shrinkage and selection operator (LASSO) regression for dimensionality reduction, followed by multivariate Cox regression to identify the final prognostic gene set and their coefficients. A patient‐specific risk score was calculated as follows: RiskScore = ∑(*β*_i × Exp_i), in which *β*_i denotes the regression coefficient of gene i, and Exp_i represents its expression level. Patients were stratified into high‐ and low‐risk groups using an optimal cutoff determined by the “survminer” package [38]. The prognostic performance was assessed by Kaplan–Meier (KM) survival analysis and time‐dependent receiver operating characteristic (ROC) curves.

### 2.7. Immune Microenvironment Analysis

Enrichment scores for 28 immune‐cell subsets were calculated using ssGSEA implemented in the GSVA package to characterize their relative enrichment and the tumor immune microenvironment of each sample [39].

### 2.8. Drug Sensitivity Analysis

Drug sensitivity was evaluated using the pRRophetic package [40], which generated in silico predicted half‐maximal inhibitory concentration (IC50) values for candidate antitumor compounds in the TCGA‐CRC cohort. Spearman correlation analysis was used to assess associations between the RiskScore and predicted IC50 values. *p* values from the drug‐wise correlation analyses were adjusted for multiple testing using the Benjamini–Hochberg method, and an adjusted *p* < 0.05 was considered significant. Lower predicted IC50 values were interpreted as greater predicted sensitivity; these estimates were not considered equivalent to observed clinical response.

### 2.9. Cell Culture and Transfection

Human COAD cell line SW1116 (*Homo sapiens*, male, COAD; official name: SW1116; RRID: CVCL_0544) and human normal colonic epithelial cells NCM460 (*H. sapiens*; colon epithelium; official name: NCM460; RRID: CVCL_0460) were used in this work. SW1116 cells were obtained from the American Type Culture Collection (ATCC, CCL‐233, Manassas, Virginia, United States) in March 2023 and NCM460 cells were obtained from Immocell (IM‐H445, Xiamen, China) in April 2023. All cell lines were authenticated by short tandem repeat (STR) profiling (GenePharma, Shanghai, China) and confirmed to be free of mycoplasma contamination (last tested in June 2024). According to the ICLAC and Cellosaurus databases, both SW1116 and NCM460 are not reported as misidentified or contaminated cell lines. Cells were cultured in DMEM (PM150210, Procell, Wuhan, China) containing 10% fetal bovine serum (164210, Procell) and 1% penicillin–streptomycin (PB180120), at 37°C, in a humidified incubator containing 5% CO_2_, and then passaged with 70%–80% confluence using Trypsin–EDTA. Small interfering RNAs directly targeting CD36 (si‐CD36) and a nontargeting negative control siRNA were purchased from GenePharma (Shanghai, China) for gene silencing. The sequences were si‐NC (5 ^′^‐UUCUCCGAACGUGUCACGUTT‐3 ^′^), si‐CD36#1 (5 ^′^‐GGCUGUGUUUGGAGGUAUUCUTT‐3 ^′^) and si‐CD36#2 (5 ^′^‐CCCUGUUACUACCACAGUUTT‐3 ^′^). Then, cultures containing SW1116 were plated in ~60%–70% confluence at transfection point. siRNAs were transfected by using Lipofectamine 3000 (11668027, Invitrogen, Carlsbad, California, United States) (as per the manufacturer′s instructions), and siRNA–lipid complexes were prepared in Opti‐MEM for addition into cells (final siRNA concentration was 50 nM), after 6 h the medium content was replaced with fresh complete medium, and cells were collected for 24−48 h after transfection for reverse transcription quantitative PCR (RT‐qPCR) to verify efficiency of knockdown. Functional assays (CCK‐8, wound healing, and Transwell invasion) were performed 24 h after transfection unless stated otherwise.

### 2.10. RT‐qPCR

Total RNA was extracted from cells using TRIzol reagent (15596026CN; Invitrogen, Carlsbad, California, United States), and its concentration/purity was measured with a NanoDrop spectrophotometer (Thermo Fisher Scientific, United States). Complementary DNA was synthesized with the Beyotime Reverse Transcription Kit (D7178S; Beyotime, Shanghai, China). Quantitative real‐time PCR was performed on an Applied Biosystems platform (Applied Biosystems, Foster City, California, United States) using SYBR Green qPCR Mix (D7260; Beyotime, China), with the following program: 95°C for 10 min; 30 cycles of 95°C for 15 s, 60°C for 30 s, 72°C for 1 min; and 72°C for 5 min. Relative mRNA expression was calculated via the 2^−*Δ* *Δ*Ct^ method with GAPDH as the reference gene [41]. Primer sequences are listed in Table 1. All samples were run in triplicate alongside no‐template controls.

**Table 1:** The primers utilized in this study.

Gene	Accession No.	Primers (5 ^′^‐3 ^′^)	
		Forward	Reverse
*CD36*	NM_000072	CAGGTCAACCTATTGGTCAAGCC	GCCTTCTCATCACCAATGGTCC
*CPA3*	NM_001870	ATCGGCACTGACCTCAACAGGA	CTCTTTCTCGGACTCTGGTGCA
*FCRL2*	NM_030764	TGTGCCTATCCAGAGCAAGGTG	TCACAGTGAAGCTCCAGCAGGT
*POU2F2*	NM_002698	TCCTGGAGAAGTGGCTCAACGA	ATGCTGGTCCTCTTCTTGCGTC
*S100B*	NM_006272	GAAGAAATCCGAACTGAAGGAGC	TCCTGGAAGTCACATTCGCCGT
*GAPDH*	NM_002046	GTCTCCTCTGACTTCAACAGCG	ACCACCCTGTTGCTGTAGCCAA

### 2.11. Cell Viability Assay

Cells were seeded in 96‐well plates (2 × 10^3^ cells/well) and cultured in complete DMEM (10% FBS, 1% penicillin–streptomycin) under standard conditions (at 37°C, 5% CO_2_) for 48 h. Cell viability was then assessed using CCK‐8 assay (C0037, Beyotime, China). After adding 10 *μ*L of CCK‐8 solution, each well was incubated for 4 h. Next, a microplate reader (Thermo Fisher Scientific, United States) was employed to read the absorbance at 450 nm. Cell viability (%) was then calculated from the absorbance values using the formula: Percent viability = (OD450(sample) − OD450(blank))/(OD450(si − NC) − OD450(blank)) × 100*%*, with background correction and normalization to control. All conditions included ≥ 3 technical replicates and were independently repeated three times at least.

### 2.12. Wound‐Healing Assay

Cells were cultured in 6‐well plates (2‐mL complete medium/well) at 37°C with 5% CO_2_ until reaching confluence. A straight scratch was then made using a sterile 200‐*μ*L pipette tip, and the wells were rinsed twice with PBS to remove dislodged cells. Serum‐free DMEM was subsequently added. Phase‐contrast images were acquired at 0 and 48 h using a CKX53 inverted microscope (Evident Corporation, Tokyo, Japan). The wound closure rate was determined as the percentage decrease in the average wound width at 48 h relative to the initial width. All experiments included technical triplicates and were independently reproduced a minimum of three times.

### 2.13. Transwell Assay

Transwell inserts (Corning Costar, Corning, New York, United States) were first coated on the upper membrane with 30 *μ*L of Matrigel and allowed to polymerize. Cells were subsequently seeded into the upper chambers, whereas the lower chamber was added with medium containing FBS. After incubation at 37°C for 48 h, noninvading cells were carefully removed, whereas 4% paraformaldehyde was used to fix the invaded cells, which were colored by crystal violet for 10 min, and then imaged and enumerated using a CKX53 inverted microscope (Evident Corporation, Tokyo, Japan).

### 2.14. Statistical Analysis

Statistical analyses were performed using R Version 3.6.0 and GraphPad Prism Version 9.5.0. The Wilcoxon rank‐sum test, Spearman correlation, log‐rank test, Student′s *t*‐test, and one‐ or two‐way ANOVA followed by appropriate multiple‐comparisons tests were used as applicable. *p* values from drug‐wise correlation analyses were adjusted using the Benjamini–Hochberg method. Data are presented as the mean ± SEM or SD, as indicated. *p* < 0.05 was considered statistically significant.

## 3. Results

### 3.1. Identification of Chemotherapy Response–Associated Genes in GSE104645

Differential expression analysis between chemotherapy‐sensitive and resistant cases in GSE104645 identified 118 genes at *p* < 0.05, including 51 genes with higher expression in the sensitive group and 67 genes with higher expression in the resistant group. The 51 genes upregulated in the sensitive group were used as the feature set for calculating the drug‐sensitivity gene‐set score by ssGSEA.

### 3.2. Distribution of MMRG Scores and Drug Sensitivity Scores, and Prognostic Value Analysis

The ssGSEA of the TCGA‐CRC cohort indicated that tumor tissues exhibited significantly lower MMRG scores than normal adjacent tissues (Figure 1A), indicating a general reduction in the activity of this MMRG set in tumors. In contrast, drug sensitivity scores were significantly higher in the tumor group (Figure 1B). Further KM survival analysis demonstrated that patients with high MMRG scores had significantly better OS than those with low scores (Figure 1C), indicating that the MMRG score was associated with OS in this cohort. In contrast, no significant difference in OS was detected between the high‐ and low drug–sensitivity score groups (log − rank *p* = 0.71; Figure 1D). Thus, although the score differed between tumor and normal samples, it was not associated with OS in the TCGA‐CRC cohort.

**Figure 1:**
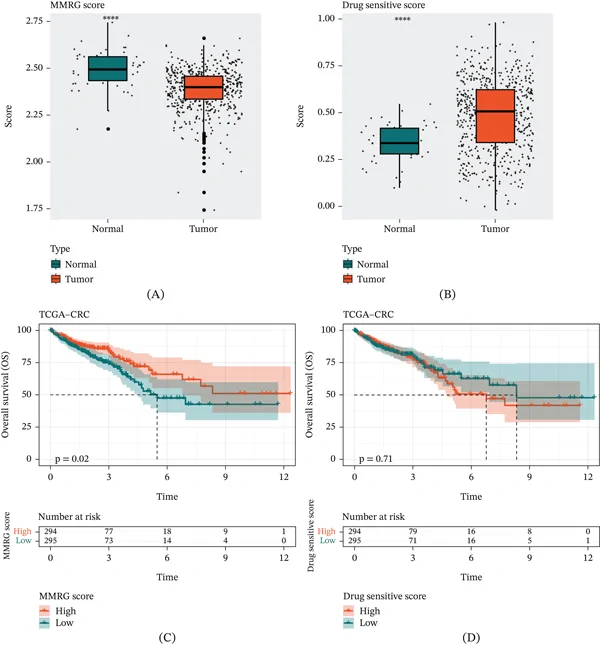
Distribution and prognostic value of MMRG and drug sensitivity scores in the TCGA‐CRC cohort. (A) Boxplots for the distribution of MMRG scores between normal colonic tissues and CRC tumor tissues in the TCGA‐CRC cohort. (B) Boxplots comparing the distribution of drug sensitivity scores between normal and tumor tissues in the TCGA‐CRC cohort. (C) KM curves for OS in patients stratified into high‐ and low‐MMRG score groups by the median MMRG score. (D) KM curves for OS in patients stratified into high‐ and low‐drug sensitivity score groups based on the median score. Log − rank *p* = 0.71,  ^∗∗∗∗^ *p* < 0.001.

### 3.3. Identification of Key Gene Modules Associated With MMRG and Drug Sensitivity Scores by WGCNA

Following outlier removal via hierarchical clustering, a coexpression network was constructed to identify MMRG‐ and drug sensitivity‐related gene modules. Using a soft‐threshold power (*β* = 18) to satisfy scale‐free topology (Figure 2A), dynamic tree cutting identified eight distinct coexpression modules (Figure 2B,C), whereas genes that could not be assigned to any specific cluster were grouped into the grey module. Correlation analysis between module eigengenes and the MMRG and drug‐sensitivity scores indicated that the green module showed significant negative associations with both the MMRG score (*r* = −0.39, *p* = 1.64 × 10 − 22) and the drug‐sensitivity score (*r* = −0.30, *p* = 1.98 × 10 − 13) and was therefore selected as the module jointly associated with both traits (Figure 2E). The distribution of gene numbers across the identified modules is presented in Figure 2D.

**Figure 2:**
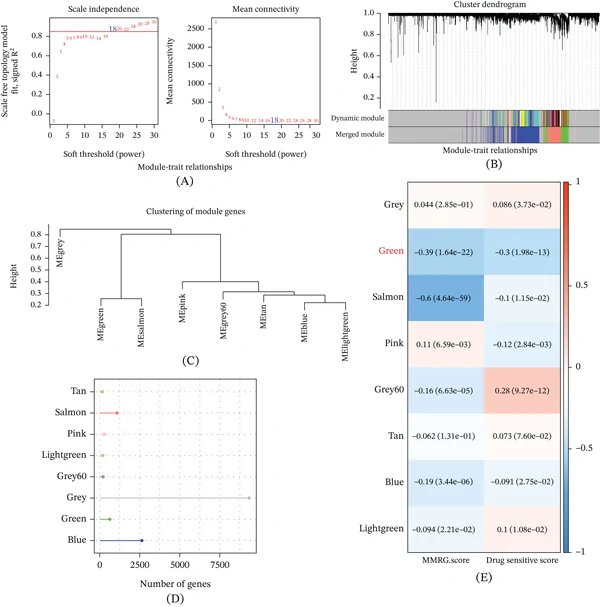
WGCNA construction and key coexpression modules associated with MMRG and drug sensitivity scores. (A) Analysis of scale‐free topology fit index (left) and mean connectivity (right) across a range of soft‐thresholding powers (*β*) for network construction. (B) Based on the topological overlap dissimilarity measure (1 − TOM), gene dendrogram generated by hierarchical clustering, with different colors representing the initial gene modules. (C) Dendrogram of module eigengenes showing the hierarchical clustering relationships among the coexpression modules and the results of module merging. (D) Bar plot displaying the number of genes contained in each coexpression module, including the grey module, which comprises genes not assigned to any specific module. (E) Heatmap showing the correlations between module eigengenes and the phenotypic traits (MMRG score and drug sensitivity score).

### 3.4. Functional Enrichment Analysis of DEGs and Intersection With the Green Module

Intersection of the TCGA‐CRC tumor‐versus‐normal DEGs with the green‐module genes yielded 287 overlapping genes (Figure 3A,B). Functional enrichment analysis using “clusterProfiler” package revealed that these genes were closely linked to immune processes (e.g., T cell activation and leukocyte proliferation) in GO‐BP terms (Figure 3C), and to pathways like cell adhesion molecules and chemokine signaling in KEGG pathways (Figure 3D). These results suggested their key roles in tumor immune regulation and intercellular interaction.

**Figure 3:**
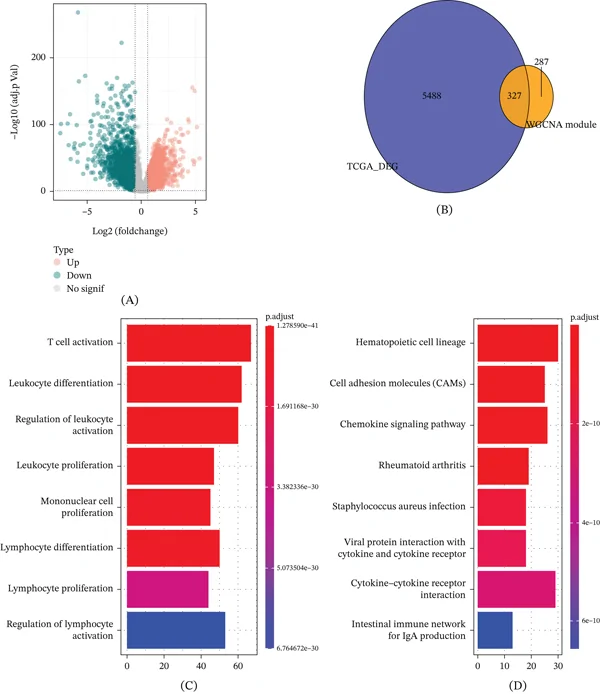
Differentially expressed genes in TCGA‐CRC and functional enrichment of the intersection with the green module. (A) Volcano plot of DEGs between normal and tumor tissues in the TCGA‐CRC cohort. (B) Venn diagram showing the overlap between TCGA‐CRC DEGs and genes from the WGCNA green module. (C) GO BP enrichment analysis of the intersecting genes. (D) KEGG pathway enrichment analysis of the intersecting genes.

### 3.5. Risk Model

The TCGA‐CRC cohort was randomly split 1:1 into training and testing sets for model development and validation. In the training set, a five‐gene prognostic signature (CD36, CPA3, FCRL2, POU2F2, S100B, *p* < 0.05) was derived via sequential Cox and LASSO regression (Figure 4A), yielding the risk score:

**Figure 4:**
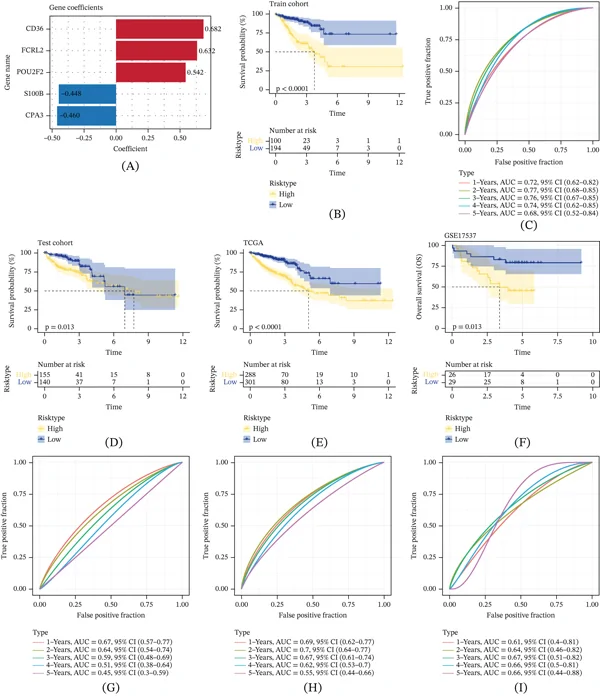
Development of the five‐gene mitochondrial metabolism–related risk model and validation (A) Bar plot showing the regression coefficients of the five signature genes (CD36, CPA3, FCRL2, POU2F2, and S100B) in the multivariate Cox model derived from the TCGA training cohort. (B) KM curves for patients in high‐ and low‐risk groups in the TCGA training cohort (C) Time‐dependent ROC curves of the RiskScore for predicting 1‐, 2‐, 3‐, 4‐, and 5‐year OS in the TCGA training cohort. (D) KM curve for the two risk groups in the TCGA testing cohort. (E) KM curve for the two risk groups in the entire TCGA‐CRC cohort. (F) KM curve for the two risk groups in the external validation cohort GSE17537. (G–I) Time‐dependent ROC curves of (G) the RiskScore for predicting 1‐, 2‐, 3‐, 4‐, and 5‐year OS in the TCGA testing cohort, (H) the entire TCGA‐CRC cohort, and (I) the GSE17537 cohort.

RiskScore = (0.682 × CD36) + (−0.460 × CPA3) + (0.632 × FCRL2) + (0.542 × POU2F2) + (−0.448 × S100B).

Using the optimal cutoff value derived from this score, patients were stratified into high‐ and low‐risk groups. KM analysis showed shorter OS in the high‐risk group in the training, testing, entire TCGA‐CRC, and GSE17537 cohorts (Figure 4B,D–F). Time‐dependent AUC values ranged from 0.68 to 0.77 in the training cohort, 0.45 to 0.67 in the testing cohort, 0.55 to 0.70 in the entire TCGA‐CRC cohort, and 0.61 to 0.67 in GSE17537 (Figure 4C,G–I). These results indicate moderate prognostic discrimination that varied across cohorts and follow‐up time points, particularly at later time points in the testing cohort.

### 3.6. Subgroup Survival Associations of the RiskScore

Stratified survival analysis revealed that the risk score demonstrated strong prognostic discrimination ability across different clinical subgroups (Figure 5A–F). In early‐stage patients (Stage I + II), the high‐risk group showed more unfavorable OS than the low‐risk group (*p* = 0.00088, Figure 5A). Similarly, in advanced‐stage patients (Stage III + IV), the high‐risk group also exhibited significantly poorer prognosis (*p* = 0.0017, Figure 5B). Age‐stratified analysis showed that, regardless of age ≤ 67 years (*p* = 0.014, Figure 5C) or age > 67 years (*p* < 0.0001, Figure 5D), high‐risk patients had worse survival outcomes. Gender‐based stratification further confirmed similar trends in both female (*p* < 0.0001, Figure 5E) and male (*p* = 0.013, Figure 5F) patients, where significantly lower OS was detected in the high‐risk group. Collectively, the RiskScore remained associated with OS within the examined stage, age, and sex subgroups. However, these stratified analyses do not establish that the RiskScore is prognostically independent of, or adds incremental value beyond, conventional clinicopathological variables.

**Figure 5:**
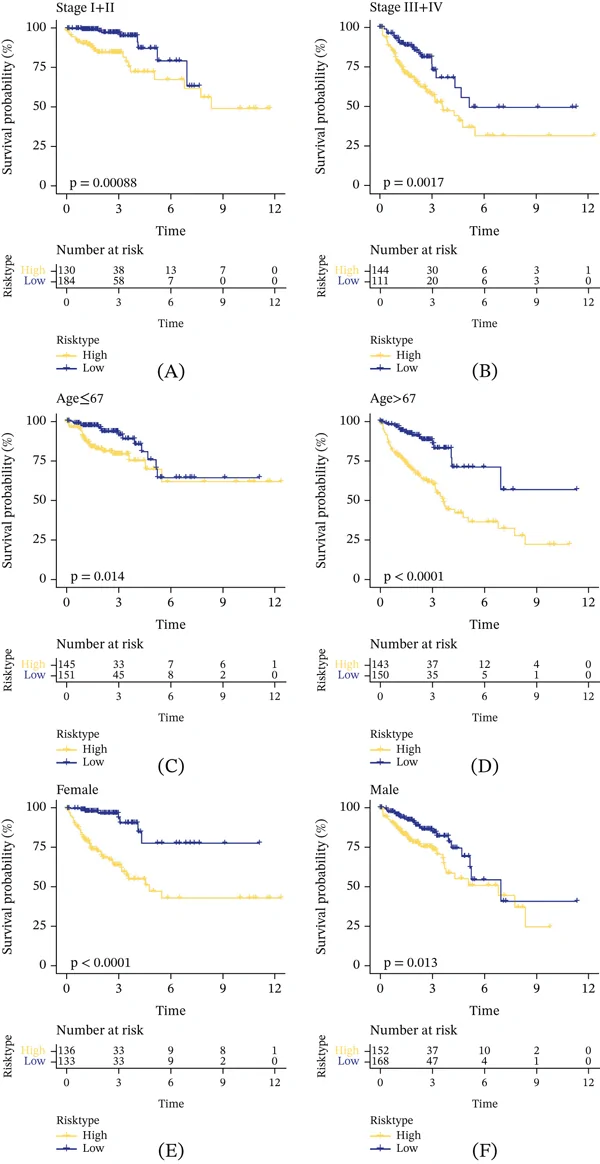
Stratified survival analysis of the RiskScore in different clinical subgroups of the TCGA‐CRC cohort. (A,B) KM curve for the two risk groups in patients with early‐stage ((A) Stage I + II) and advanced‐stage ((B) Stage III + IV) colorectal cancer. (C,D) KM curve for (C) patients aged ≤ 67 years and (D) patients aged > 67 years, stratified by RiskScore. (E,F) KM curve comparing high‐ and low‐risk groups in (E) female and (F) male patients, respectively.

### 3.7. Analysis of the Relationship Between Risk Score, Immune Infiltration Characteristics, and Drug Sensitivity

Comparative analyses revealed distinct immune and drug‐response profiles between risk groups. The high‐ and low‐risk groups exhibited differences in the ssGSEA enrichment scores of several immune‐cell subsets, including activated and memory CD8^+^ T cells, *γδ* T cells, neutrophils, mast cells, macrophages, and myeloid‐derived suppressor cells (Figure 6A). Because both antitumor and potentially immunosuppressive cell populations differed between the groups, these findings were interpreted as evidence of altered immune composition rather than a uniformly immunosuppressive state. After Benjamini–Hochberg correction, the RiskScore was negatively correlated with the pRRophetic‐predicted IC50 values of pyrimethamine, MS.275, AZ628, and bortezomib, whereas a positive correlation was observed for cisplatin (Figure 6B). These correlations were modest and based on algorithm‐derived IC50 estimates; therefore, they should be interpreted as exploratory hypotheses rather than evidence of actual clinical response or treatment benefit.

**Figure 6:**
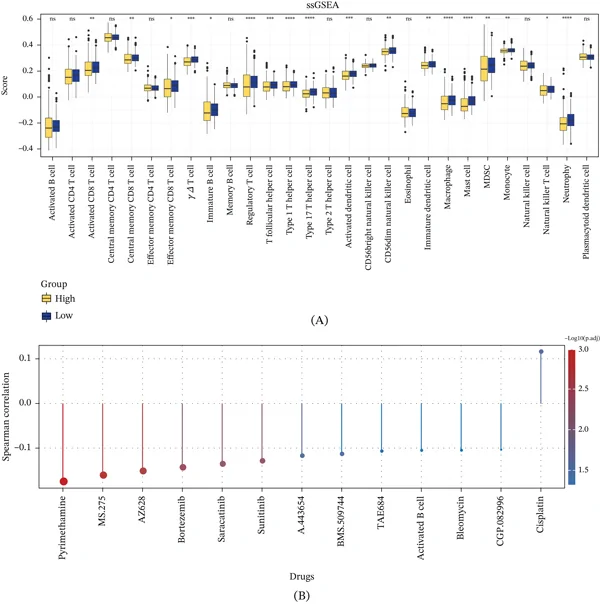
Associations of the RiskScore with immune‐cell enrichment and in silico–predicted drug sensitivity in the TCGA‐CRC cohort. (A) Boxplots comparing ssGSEA enrichment scores for 28 immune‐cell subsets between the high‐ and low‐risk groups. Group comparisons were performed using the Wilcoxon rank‐sum test. (B) Lollipop plot showing Spearman correlations between the RiskScore and pRRophetic‐predicted IC50 values. Point position represents Spearman′s *ρ*, point size represents |*ρ*|, and point color represents −log10 of the Benjamini–Hochberg‐adjusted *p* value. Negative coefficients indicate lower predicted IC50 values at higher RiskScores. Adjusted *p* < 0.05 was considered statistically significant.  ^∗∗∗∗^ *p* < 0.0001,  ^∗∗∗^ *p* < 0.001,  ^∗∗^ *p* < 0.01,  ^∗^ *p* < 0.05.

### 3.8. Experimental Validation of CD36 Expression and Its Functional Role in SW1116 Cells

To experimentally validate the risk‐model–derived genes, the mRNA expression of CD36, CPA3, FCRL2, POU2F2, and S100B was first quantified in NCM460 and SW1116 cells by RT‐qPCR. CD36, POU2F2, and S100B were markedly upregulated in SW1116 cells compared with NCM460 cells, whereas CPA3 and FCRL2 showed no significant difference between the two cell lines (Figure 7A). To investigate the biological effects of CD36, two siRNAs (si‐CD36#1 and si‐CD36#2) were used, and knockdown efficiency was confirmed by RT‐qPCR. Both siRNAs significantly reduced CD36 mRNA expression, with si‐CD36#1 exhibiting the most pronounced suppression (Figure 7B). CCK‐8 assays showed that CD36 silencing markedly impaired the proliferative capacity of SW1116 cells, as reflected by reduced OD450 values at 24, 48, and 72 h in the si‐CD36#1 group compared with the si‐NC group (Figure 7C). Consistently, wound‐healing assays demonstrated that knockdown of CD36 significantly inhibited cell migration, as indicated by a reduced wound‐closure rate at 48 h in si‐CD36#1–transfected cells (Figure 7D). Transwell invasion assays further revealed that CD36 silencing markedly decreased the number of invading SW1116 cells (Figure 7E). Together, these results indicate that CD36 expression was higher in SW1116 than in NCM460 cells and that CD36 knockdown reduced SW1116‐cell proliferation, migration, and invasion. Because mitochondrial function was not directly measured, these results do not establish that the observed phenotypes depend on mitochondrial metabolic reprogramming. CD36 was selected for functional investigation because it showed increased expression in SW1116 cells, carried a positive coefficient in the prognostic model, and had the most direct reported relationship with fatty acid uptake and metabolic adaptation.

**Figure 7:**
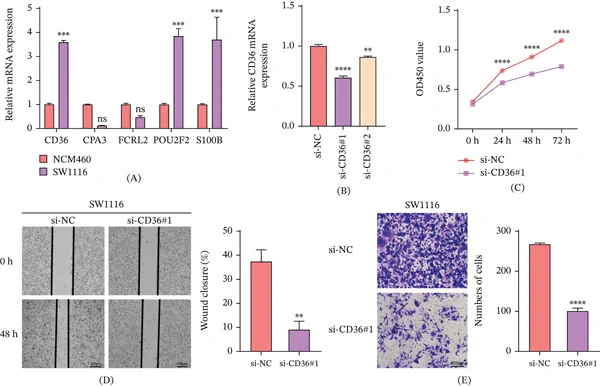
Experimental evaluation of the five model genes and phenotypic effects of CD36 knockdown in SW1116 cells. (A) Relative mRNA expression of CD36, CPA3, FCRL2, POU2F2, and S100B in normal human colonic epithelial cells (NCM460) and colon adenocarcinoma cells (SW1116), as determined by RT‐qPCR. (B) Validation of CD36 knockdown efficiency in SW1116 cells transfected with si‐NC, si‐CD36#1, or si‐CD36#2, measured by RT‐qPCR. (C) Proliferation of SW1116 cells transfected with si‐NC or si‐CD36#1, evaluated at 0, 24, 48, and 72 h using the CCK‐8 assay. (D) Wound‐healing assay illustrating the effect of CD36 silencing on SW1116 cell migration at 0 and 48 h, with quantitative analysis of wound‐closure rates. (E) Transwell invasion assay demonstrating the influence of CD36 knockdown on the invasive behavior of SW1116 cells, with representative micrographs and quantification of invading cells. Scale bars, 200 *μ*m (D,E). ns = not significant,  ^∗∗∗∗^ *p* < 0.0001,  ^∗∗∗^ *p* < 0.001,  ^∗∗^ *p* < 0.01.

## 4. Discussion

CRC remains a major cause of cancer‐related mortality, and intrinsic or acquired treatment resistance limits long‐term survival. The present study integrated MMRGs, a chemotherapy response–derived transcriptional score, and multiple CRC transcriptomic cohorts to construct a five‐gene RiskScore. Higher MMRG scores and lower RiskScores were associated with better OS, whereas the drug‐sensitivity gene‐set score itself was not significantly associated with OS. The five‐gene model separated survival outcomes in internal and external cohorts, although its time‐dependent discrimination varied. The RiskScore was also associated with altered immune‐cell enrichment and exploratory correlations with predicted drug IC50 values. CD36 knockdown reduced malignant phenotypes in SW1116 cells, but the experiments did not directly evaluate mitochondrial function. These findings therefore support a mitochondria‐related prognostic framework while avoiding conclusions regarding clinical treatment selection or a proven mitochondria‐dependent CD36 mechanism.

Recent studies have described mitochondrial dynamics, lipid metabolism, ferroptosis‐related, and machine learning–derived mitochondrial signatures in CRC [42–47]. The present model differs by incorporating a drug response–derived ssGSEA trait from GSE104645 into WGCNA before survival‐model construction and by evaluating immune‐cell enrichment and predicted IC50 correlations alongside external survival validation. Nevertheless, the current analysis did not directly compare the RiskScore with a clinicopathological reference model or test changes in concordance, calibration, or decision‐curve benefit. The subgroup analyses demonstrate survival associations within stage, age, and sex strata but do not establish independent or incremental prognostic value. Similarly, the pRRophetic analysis reflects in silico drug‐sensitivity estimates rather than observed chemotherapy benefit.

At the biological level, the enrichment and immune‐cell analyses suggest a potential mitochondrial metabolism–immune microenvironment link underlying the prognostic associations of the signature. The 287 intersecting genes derived from the MMRG‐associated module were enriched in T cell activation, leukocyte proliferation, lymphocyte differentiation, and cell adhesion molecules and chemokine signaling, pointing to a tight coupling between mitochondrial‐related transcriptional programs and immune regulation as well as cell–cell and cell–matrix interactions. Consistent with this, high‐risk tumors exhibited reduced infiltration of activated and memory CD8^+^ T cells, *γδ* T cells, neutrophils, mast cells, macrophages, and myeloid‐derived suppressor cells, indicating broad differences in immune cell–related transcriptional enrichment between the risk groups. Because the high‐risk group also showed lower enrichment of myeloid‐derived suppressor cells and other immune populations with context‐dependent functions, the results do not support classification of the high‐risk group as uniformly immune‐suppressed or “cold.” These observations align with accumulating evidence that mitochondrial metabolism governs T cell activation, differentiation, effector function, and exhaustion, and that mitochondrial dysfunction or altered redox signaling can promote T‐cell dysfunction within the TME [48, 49]. Recent reviews have further emphasized that mitochondrial lipid metabolism and organelle crosstalk shape antitumor immunity and immunosurveillance, and that mitochondrial rewiring in cancer cells can constrain nutrient availability and generate toxic metabolite gradients that selectively impair effector T cell responses [50–52].

Among the five model genes, CD36 emerged as a representative candidate that bridges mitochondrial metabolism, lipid handling, and malignant behavior. CD36 is a fatty acid translocase that mediates uptake of long‐chain fatty acids and contributes to lipid storage and *β*‐oxidation, thereby supporting energy production and membrane biogenesis in metabolically stressed cancer cells. Multiple recent studies in CRC and other solid tumors have shown that dysregulated fatty acid metabolism—including enhanced uptake via CD36—promotes proliferation, EMT, metastatic dissemination, and resistance to chemotherapy [3, 53, 54]. In CRC models, CD36 upregulation has been linked to increased fatty acid uptake and metabolic reprogramming, correlating with more aggressive phenotypes and poor prognosis, whereas broader analyses of host and microbiome lipid metabolism in CRC have also highlighted CD36 as a key node at the interface between dietary lipids, microbial metabolites, and tumor progression [55–57]. The elevated expression of CD36 in SW1116 cells and the reduction in proliferation, migration, and invasion after CD36 knockdown are consistent with a tumor‐promoting role in this cellular context. CD36‐mediated fatty acid uptake could plausibly influence mitochondrial substrate utilization; however, this mechanism was not tested directly. Accordingly, the present experiments support CD36 as a candidate for further mechanistic investigation but do not establish CD36‐dependent fatty acid oxidation, mitochondrial reprogramming, or therapeutic vulnerability in high‐risk CRC.

The other four model genes may connect the RiskScore to immune‐cell composition and tumor‐cell state rather than representing direct mitochondrial regulators. CPA3 is a mast‐cell protease marker, and single‐cell analyses have identified CPA3‐high activated mast‐cell populations in CRC; its negative Cox coefficient is directionally consistent with the lower mast‐cell ssGSEA score observed in the high‐risk group [58]. FCRL2 is an immunoregulatory receptor expressed predominantly in B cells and can inhibit B cell receptor signaling [59]. Its positive model coefficient may therefore reflect a specific immune‐cell state or cellular‐composition signal, although a direct role in CRC mitochondrial metabolism has not been established. POU2F2, also known as OCT2, has been implicated in escape from oncogene‐induced senescence during CRC progression, and its activity has been associated with poorer survival [60], providing a possible explanation for its positive coefficient and elevated expression in SW1116 cells. S100B can promote proliferation and invasiveness of COAD cells through RAGE/p38/Akt/mTOR–related signaling [61]; however, its negative multivariable Cox coefficient despite higher expression in SW1116 cells illustrates that model coefficients should not be interpreted as isolated causal effects. Collectively, these genes may capture a mixture of tumor‐intrinsic and immune‐microenvironmental signals. With the exception of the plausible metabolic link provided by CD36, direct mitochondrial functions of CPA3, FCRL2, POU2F2, and S100B remain to be demonstrated.

Several limitations should be acknowledged. First, the analyses were retrospective and based primarily on bulk transcriptomic datasets. Heterogeneity in follow‐up duration and clinical annotation within TCGA may introduce bias, and no multivariable clinicopathological comparison was performed to establish the independent or incremental value of the RiskScore. Second, the external GSE17537 cohort contained only 55 patients, limiting the precision and generalizability of external validation. Third, immune‐cell enrichment and drug sensitivity were inferred computationally. Although Benjamini–Hochberg correction was applied to drug‐wise correlations, pRRophetic‐predicted IC50 values were not validated against actual treatment responses in GSE104645 or another treatment‐annotated cohort and should not be considered evidence of clinical drug benefit. Fourth, functional validation was limited to CD36 in a single CRC cell line. CPA3, FCRL2, POU2F2, and S100B were not functionally investigated, and no direct measurements of mitochondrial respiration, membrane potential, ATP production, ROS, or fatty acid oxidation were performed after CD36 knockdown. Consequently, the current experiments cannot demonstrate that the CD36‐associated phenotypes are mediated by mitochondrial metabolic reprogramming. Validation in additional CRC cell lines, primary models, prospective cohorts, and direct mitochondrial assays is required.

## 5. Conclusions

A five‐gene mitochondrial metabolism–related RiskScore comprising CD36, CPA3, FCRL2, POU2F2, and S100B was associated with OS in internal and external CRC cohorts and showed similar survival associations across several clinical subgroups. The RiskScore was also associated with altered immune‐cell enrichment and exploratory in silico drug‐sensitivity estimates. CD36 knockdown reduced proliferation, migration, and invasion in SW1116 cells, although a mitochondria‐dependent mechanism was not examined. The signature should therefore be regarded as a candidate prognostic framework requiring prospective clinical validation and additional mechanistic investigation rather than as an established tool for treatment selection.

**Nomenclature**
AUC: area under the curve
BP: biological process
CAMs: cell adhesion molecules
CCK‐8: Cell Counting Kit‐8
CD36: cluster of differentiation 36
COAD: colon adenocarcinoma
CR: complete response
CRC: colorectal cancer
DEGs: differentially expressed genes
DMEM: Dulbecco′s Modified Eagle′s Medium
EGFR: epidermal growth factor receptor
FBS: fetal bovine serum
FCRL2: Fc receptor‐like 2
FOLFIRI: 5‐fluorouracil, leucovorin, and irinotecan
FOLFOX: 5‐fluorouracil, leucovorin, and oxaliplatin
GAPDH: glyceraldehyde‐3‐phosphate dehydrogenase
GEO: Gene Expression Omnibus
GO: Gene Ontology
IC50: half‐maximal inhibitory concentration
KEGG: Kyoto Encyclopedia of Genes and Genomes
KM: Kaplan–Meier
MDSC: myeloid‐derived suppressor cell
MMRGs: mitochondrial metabolism–related genes
NC: negative control
NCM460: normal colonic epithelial cell line 460
OS: overall survival
PBS: phosphate‐buffered saline
PD: progressive disease
POU2F2: POU class 2 homeobox 2
PR: partial response
READ: rectal adenocarcinoma
RiskScore: mitochondrial metabolism–related gene–based risk score
ROC: receiver operating characteristic
RT‐qPCR: reverse transcription quantitative polymerase chain reaction
S100B: S100 calcium‐binding protein B
SD: stable disease
siRNA: small interfering RNA
ssGSEA: single‐sample gene set enrichment analysis
SW1116: human colon adenocarcinoma cell line SW1116
TCGA: The Cancer Genome Atlas
TME: tumor microenvironment
WGCNA: weighted gene coexpression network analysis

## Author Contributions

All authors contributed to this present work: J.T. and J.X. designed the study, D.Y. acquired the data, and J.X. interpreted the data. Z.W. drafted the manuscript, and Y.X. completed the experiment and revised the manuscript.

## Funding

This study was supported by the Zhejiang Province Traditional Chinese Medicine Science and Technology Plan (2026ZL0123) and Key Discipline of Ningbo No.2 Hospital, Department of Hematology and Oncology (2023‐Y08).

## Disclosure

All authors read and approved the manuscript.

## Ethics Statement

Ethical approval was not required for this study because it did not involve any human experiments.

## Consent

The authors have nothing to report.

## Conflicts of Interest

The authors declare no conflicts of interest.

## Data Availability

The datasets generated and/or analyzed during the current study are available in the GSE17537 repository (https://www.ncbi.nlm.nih.gov/geo/query/acc.cgi?acc=GSE17537) and GSE104645 repository (https://www.ncbi.nlm.nih.gov/geo/query/acc.cgi?acc=GSE104645).

## References

[1] Morgan E. , Arnold M. , Gini A. , Lorenzoni V. , Cabasag C. J. , Laversanne M. , Vignat J. , Ferlay J. , Murphy N. , and Bray F. , Global Burden of Colorectal Cancer in 2020 and 2040: Incidence and Mortality Estimates From Globocan, Gut. (2023) 72, no. 2, 338–344, 10.1136/gutjnl-2022-327736, 36604116. 36604116

[2] Xu Z. , Yu Y. , Ni H. , Sun W. , and Kuang Y. , Linc01836 Promotes Colorectal Cancer Progression and Functions as Cerna to Target Slc17a9 by Sponging Mir-1226-3p, Protein & Peptide Letters. (2024) 31, no. 1, 43–60, 10.2174/0109298665248028231122064831, 38058092. 38058092

[3] Jiang T. , Zheng J. , Li N. , Li X. , He J. , Zhou J. , Sun B. , and Chi Q. , Dissecting the Mechanisms of Intestinal Immune Homeostasis by Analyzing T-Cell Immune Response in Crohn′s Disease and Colorectal Cancer, Current Gene Therapy. (2024) 24, no. 5, 422–440, 10.2174/0115665232294568240201073417, 38682449. 38682449

[4] Lopes S. R. , Martins C. , Santos I. C. , Teixeira M. , Gamito É. , and Alves A. L. , Colorectal Cancer Screening: A Review of Current Knowledge and Progress in Research, World Journal of Gastrointestinal Oncology. (2024) 16, no. 4, 1119–1133, 10.4251/wjgo.v16.i4.1119, 38660635. PMC11037045 38660635

[5] Balboa-Barreiro V. , Pértega-Díaz S. , García-Rodríguez T. , González-Martín C. , Pardeiro-Pértega R. , Yáñez-González-Dopeso L. , and Seoane-Pillado T. , Colorectal Cancer Recurrence and Its Impact on Survival After Curative Surgery: An Analysis Based on Multistate Models, Digestive and Liver Disease. (2024) 56, no. 7, 1229–1236, 10.1016/j.dld.2023.11.041, 38087671. 38087671

[6] Xie Y. H. , Chen Y. X. , and Fang J. Y. , Comprehensive Review of Targeted Therapy for Colorectal Cancer, Signal Transduction and Targeted Therapy. (2020) 5, no. 1, 10.1038/s41392-020-0116-z, 32296018. PMC7082344 32296018

[7] Shinozaki E. , Makiyama A. , Kagawa Y. , Satake H. , Tanizawa Y. , Cai Z. , and Piao Y. , Treatment Sequences of Patients With Advanced Colorectal Cancer and Use of Second-Line Folfiri With Antiangiogenic Drugs in Japan: A Retrospective Observational Study Using an Administrative Database, PLoS One. (2021) 16, no. 2, e0246160, 10.1371/journal.pone.0246160, 33556095. PMC7870079 33556095

[8] Hammond W. A. , Swaika A. , and Mody K. , Pharmacologic Resistance in Colorectal Cancer: A Review, Therapeutic Advances in Medical Oncology. (2016) 8, no. 1, 57–84, 10.1177/1758834015614530, 26753006. PMC4699262 26753006

[9] Lubner S. J. , Uboha N. V. , and Deming D. A. , Primary and Acquired Resistance to Biologic Therapies in Gastrointestinal Cancers, Journal of Gastrointestinal Oncology. (2017) 8, no. 3, 499–512, 10.21037/jgo.2017.01.16, 28736637. PMC5506279 28736637

[10] Sartore-Bianchi A. , Loupakis F. , Argilés G. , and Prager G. W. , Challenging Chemoresistant Metastatic Colorectal Cancer: Therapeutic Strategies From the Clinic and From the Laboratory, Annals of Oncology. (2016) 27, no. 8, 1456–1466, 10.1093/annonc/mdw191, 27154421. 27154421

[11] Du Y. , Karatekin F. , Wang W. K. , Hong W. , and Boopathy G. T. , Cracking the Egfr Code: Cancer Biology, Resistance Mechanisms, and Future Therapeutic Frontiers, Pharmacological Reviews. (2025) 77, no. 5, 100076, 10.1016/j.pharmr.2025.100076, 40675056. 40675056

[12] Chen K. , Collins G. , Wang H. , and Toh J. W. T. , Pathological Features and Prognostication in Colorectal Cancer, Current Oncology. (2021) 28, no. 6, 5356–5383, 10.3390/curroncol28060447, 34940086. PMC8700531 34940086

[13] Khan A. U. , Jasim Y. H. , Shahid K. , Saidov J. , Atamuratova Z. , and Urazbaeva D. , Circulating Tumor DNA as a Predictive Biomarker for Colorectal Cancer Postsurgical Recurrence: A Systematic Review and Meta-Analysis, Clinical and Translational Oncology. (2026) 28, no. 4, 1348–1362, 10.1007/s12094-025-04073-y, 41107654. PMC13009131 41107654

[14] Liu S. , Zhang X. , Wang W. , Li X. , Sun X. , Zhao Y. , Wang Q. , Li Y. , Hu F. , and Ren H. , Metabolic Reprogramming and Therapeutic Resistance in Primary and Metastatic Breast Cancer, Molecular Cancer. (2024) 23, no. 1, 10.1186/s12943-024-02165-x, 39574178. PMC11580516 39574178

[15] Ohshima K. and Morii E. , Metabolic Reprogramming of Cancer Cells During Tumor Progression and Metastasis, Metabolites. (2021) 11, no. 1, 10.3390/metabo11010028, 33401771. PMC7824065 33401771

[16] Ohshima K. , Oi R. , Nojima S. , and Morii E. , Mitochondria Govern Histone Acetylation in Colorectal Cancer, Journal of Pathology. (2022) 256, no. 2, 164–173, 10.1002/path.5818, 34698384. 34698384

[17] Kang J. and Pervaiz S. , Mitochondria: Redox Metabolism and Dysfunction, Biochemistry Research International. (2012) 2012, 896751, 10.1155/2012/896751, 22593827. PMC3347708 22593827

[18] Vakifahmetoglu-Norberg H. , Ouchida A. T. , and Norberg E. , The Role of Mitochondria in Metabolism and Cell Death, Biochemical and Biophysical Research Communications. (2017) 482, no. 3, 426–431, 10.1016/j.bbrc.2016.11.088. 28212726

[19] Zhao Z. , Mei Y. , Wang Z. , and He W. , The Effect of Oxidative Phosphorylation on Cancer Drug Resistance, Cancers. (2023) 15, no. 1, 10.3390/cancers15010062, 36612059. PMC9817696 36612059

[20] Lei Y. , Cai S. , Zhang J. K. , Ding S. Q. , Zhang Z. H. , Zhang C. D. , Dai D. Q. , and Li Y. S. , The Role and Mechanism of Fatty Acid Oxidation in Cancer Drug Resistance, Cell Death Discovery. (2025) 11, no. 1, 10.1038/s41420-025-02554-1, 40514365. PMC12166077 40514365

[21] Liu Y. , Sinjab A. , Min J. , Han G. , Paradiso F. , Zhang Y. , Wang R. , Pei G. , Dai Y. , Liu Y. , Cho K. S. , Dai E. , Basi A. , Burks J. K. , Rajapakshe K. I. , Chu Y. , Jiang J. , Zhang D. , Yan X. , Guerrero P. A. , Serrano A. , Li M. , Hwang T. H. , Futreal A. , Ajani J. A. , Solis Soto L. M. , Jazaeri A. A. , Kadara H. , Maitra A. , and Wang L. , Conserved Spatial Subtypes and Cellular Neighborhoods of Cancer-Associated Fibroblasts Revealed by Single-Cell Spatial Multi-Omics, Cancer Cell. (2025) 43, no. 5, 905–924.e6, 10.1016/j.ccell.2025.03.004, 40154487. PMC12074878 40154487

[22] Qiu X. , Wang A. , Wang J. , Zhang Z. , and Tao L. , Mitochondrial Metabolic Reprogramming in Colorectal Cancer: Mechanisms of Resistance and Future Clinical Interventions, Cell Death Discovery. (2025) 11, no. 1, 10.1038/s41420-025-02670-y, 40783392. PMC12335542 40783392

[23] Wu H. , Zhang W. , Chang J. , Wu J. , Zhang X. , Jia F. , Li L. , Liu M. , and Zhu J. , Comprehensive Analysis of Mitochondrial-Related Gene Signature for Prognosis, Tumor Immune Microenvironment Evaluation, and Candidate Drug Development in Colon Cancer, Scientific Reports. (2025) 15, no. 1, 10.1038/s41598-024-85035-2, 39979377. PMC11842742 39979377

[24] Liu C. , Li Y. , Song J. , and Zhao Y. , Development and Validation of a Machine Learning-Based Prognostic Model Using Mitochondrial Dysfunction-Related Genes for Colorectal Cancer Patients, Translational Cancer Research. (2025) 14, no. 10, 6990–7006, 10.21037/tcr-2025-1148, 41234872. PMC12605350 41234872

[25] Xie S. , Zhang J. , Sun B. , Dong X. , Wang X. , Song J. , Zhang C. , and He X. , Development of a Novel Prognostic Prediction Model Using Mitochondrial-Related Genes and Single-Cell Sequencing Data for Colorectal Carcinoma, Translational Gastroenterology and Hepatology. (2025) 10, 10.21037/tgh-25-89, 41216283. PMC12596427 41216283

[26] Choudhury F. K. , Mitochondrial Redox Metabolism: The Epicenter of Metabolism During Cancer Progression, Antioxidants. (2021) 10, no. 11, 10.3390/antiox10111838, 34829708. PMC8615124 34829708

[27] Du H. , Xu T. , Yu S. , Wu S. , and Zhang J. , Mitochondrial Metabolism and Cancer Therapeutic Innovation, Signal Transduction and Targeted Therapy. (2025) 10, no. 1, 10.1038/s41392-025-02311-x, 40754534. PMC12319113 40754534

[28] Zou W. and Green D. R. , Beggars Banquet: Metabolism in the Tumor Immune Microenvironment and Cancer Therapy, Cell Metabolism. (2023) 35, no. 7, 1101–1113, 10.1016/j.cmet.2023.06.003, 37390822. PMC10527949 37390822

[29] Steinert E. M. , Vasan K. , and Chandel N. S. , Mitochondrial Metabolism Regulation of T Cell-Mediated Immunity, Annual Review of Immunology. (2021) 39, no. 1, 395–416, 10.1146/annurev-immunol-101819-082015, 33902315. PMC10403253 33902315

[30] Plata-Gómez A. B. , Chen W. , Ho P. C. , and Ling G. S. , Mitochondrial Lipid Metabolism in Tumor Immunosurveillance and Evasion, Trends in Immunology. (2025) 46, no. 12, 766–778, 10.1016/j.it.2025.08.005, 40962643. 40962643

[31] Marx C. , Sonnemann J. , Maddocks O. D. K. , Marx-Blümel L. , Beyer M. , Hoelzer D. , Thierbach R. , Maletzki C. , Linnebacher M. , Heinzel T. , and Krämer O. H. , Global Metabolic Alterations in Colorectal Cancer Cells During Irinotecan-Induced DNA Replication Stress, Cancer Metabolism. (2022) 10, no. 1, 10.1186/s40170-022-00286-9, 35787728. PMC9251592 35787728

[32] Gao H. and Xing F. , A Novel Signature Model Based on Mitochondrial-Related Genes for Predicting Survival of Colon Adenocarcinoma, BMC Medical Informatics and Decision Making. (2022) 22, no. 1, 10.1186/s12911-022-02020-3, 36273131. PMC9587559 36273131

[33] Meng C. , Sun Y. , and Liu G. , Establishment of a Prognostic Model for Ovarian Cancer Based on Mitochondrial Metabolism-Related Genes, Frontiers in Oncology. (2023) 13, 1144430, 10.3389/fonc.2023.1144430, 37256178. PMC10226651 37256178

[34] Ritchie M. E. , Phipson B. , Wu D. , Hu Y. , Law C. W. , Shi W. , and Smyth G. K. , Limma Powers Differential Expression Analyses for Rna-Sequencing and Microarray Studies, Nucleic Acids Research. (2015) 43, no. 7, e47, 10.1093/nar/gkv007, 25605792. PMC4402510 25605792

[35] Hänzelmann S. , Castelo R. , and Guinney J. , Gsva: Gene Set Variation Analysis for Microarray and Rna-Seq Data, BMC Bioinformatics. (2013) 14, no. 1, 10.1186/1471-2105-14-7, 23323831. PMC3618321 23323831

[36] Langfelder P. and Horvath S. , Wgcna: An R Package for Weighted Correlation Network Analysis, BMC Bioinformatics. (2008) 9, no. 1, 10.1186/1471-2105-9-559, 19114008. PMC2631488 19114008

[37] Wu T. , Hu E. , Xu S. , Chen M. , Guo P. , Dai Z. , Feng T. , Zhou L. , Tang W. , Zhan L. , Fu X. , Liu S. , Bo X. , and Yu G. , Clusterprofiler 4.0: A Universal Enrichment Tool for Interpreting Omics Data, Innovation. (2021) 2, no. 3, 100141, 10.1016/j.xinn.2021.100141, 34557778. PMC8454663 34557778

[38] Vychopen M. , Güresir A. , Basaran A. E. , Güresir E. , and Wach J. , Impact of Levetiracetam Use in Glioblastoma: An Individual Patient-Level Meta-Analysis Assessing Overall Survival, Neurosurgical Review. (2024) 47, no. 1, 10.1007/s10143-024-03137-x, 39653818. PMC11628436 39653818

[39] Charoentong P. , Finotello F. , Angelova M. , Mayer C. , Efremova M. , Rieder D. , Hackl H. , and Trajanoski Z. , Pan-Cancer Immunogenomic Analyses Reveal Genotype-Immunophenotype Relationships and Predictors of Response to Checkpoint Blockade, Cell Reports. (2017) 18, no. 1, 248–262, 10.1016/j.celrep.2016.12.019, 28052254. 28052254

[40] Geeleher P. , Cox N. , and Huang R. S. , Prrophetic: An R Package for Prediction of Clinical Chemotherapeutic Response from Tumor Gene Expression Levels, PLoS One. (2014) 9, no. 9, e107468, 10.1371/journal.pone.0107468, 25229481. PMC4167990 25229481

[41] Bustin S. A. , Ruijter J. M. , van den Hoff M. J. B. , Kubista M. , Pfaffl M. W. , Shipley G. L. , Tran N. , Rödiger S. , Untergasser A. , Mueller R. , Nolan T. , Milavec M. , Burns M. J. , Huggett J. F. , Vandesompele J. , and Wittwer C. T. , Miqe 2.0: Revision of the Minimum Information for Publication of Quantitative Real-Time Pcr Experiments Guidelines, Clinical Chemistry. (2025) 71, no. 6, 634–651, 10.1093/clinchem/hvaf043, 40272429. 40272429

[42] Wang H. , Zhang K. , Wang Y. , Chen M. , and Zhang M. , A Mitochondrial Lipid Metabolism–Related Gene Signature Predicts Prognosis and Immune Landscape in Colorectal Cancer, Frontiers in Immunology. (2025) 16, 1669678, 10.3389/fimmu.2025.1669678, 41293172. PMC12640859 41293172

[43] Xie Y. H. and Jiang H. Z. , The Exploration of Mitochondrial-Related Features Helps to Reveal the Prognosis and Immunotherapy Methods of Colorectal Cancer, Cancer Reports. (2024) 7, no. 1, e1914, 10.1002/cnr2.1914, 37903487. PMC10809275 37903487

[44] Wang S. , Li Y. , Wang Z. , Geng C. , Chen P. , Li Z. , Li C. , and Bai X. , Constructing a Mitochondrial-Related Genes Model Based on Machine Learning for Predicting the Prognosis and Therapeutic Effect in Colorectal Cancer, Discover Oncology. (2025) 16, no. 1, 10.1007/s12672-025-02462-x, 40317411. PMC12049353 40317411

[45] Wu Z. , Xiao C. , Long J. , Huang W. , You F. , and Li X. , Mitochondrial Dynamics and Colorectal Cancer Biology: Mechanisms and Potential Targets, Cell Communication and Signaling. (2024) 22, no. 1, 10.1186/s12964-024-01490-4, 38302953. PMC10835948 38302953

[46] Rojas M. , Manzi M. , Madurga S. , García Velásquez F. E. , Romero M. A. , Marín S. , Cascante M. , and Maurel J. , Metabolic Plasticity Drives Specific Mechanisms of Chemotherapy and Targeted Therapy Resistance in Metastatic Colorectal Cancer, Exploration of Targeted Anti-tumor Therapy. (2025) 6, 1002337, 10.37349/etat.2025.1002337, 41018484. PMC12464553 41018484

[47] Wang H. , A Mitochondrial Ferroptosis-Related Gene Signature Predicts Prognosis and Immune Landscape in Colon Cancer, Frontiers in Medicine. (2025) 12, 1614012, 10.3389/fmed.2025.1614012, 40959429. PMC12434033 40959429

[48] Park J. , Hsueh P. C. , Li Z. , and Ho P. C. , Microenvironment-Driven Metabolic Adaptations Guiding Cd8(+) T Cell Anti-Tumor Immunity, Immunity. (2023) 56, no. 1, 32–42, 10.1016/j.immuni.2022.12.008, 36630916. 36630916

[49] Steinert E. M. , Furtado Bruza B. , Danchine V. D. , Grant R. A. , Vasan K. , Kharel A. , Zhang Y. , Cui W. , Szibor M. , Weinberg S. E. , and Chandel N. S. , Mitochondrial Respiration Is Necessary for Cd8(+) T Cell Proliferation and Cell Fate, Nature Immunology. (2025) 26, no. 8, 1267–1274, 10.1038/s41590-025-02202-x, 40670617. PMC12307223 40670617

[50] Yang M. Q. , Zhang S. L. , Sun L. , Huang L. T. , Yu J. , Zhang J. H. , Tian Y. , Han C. B. , and Ma J. T. , Targeting Mitochondria: Restoring the Antitumor Efficacy of Exhausted T Cells, Molecular Cancer. (2024) 23, no. 1, 10.1186/s12943-024-02175-9, 39563438. PMC11575104 39563438

[51] Gamal W. , Mediavilla-Varela M. , Kunta V. , Sahakian E. , and Pinilla-Ibarz J. , Impact of Mitochondrial Metabolism on T-Cell Dysfunction in Chronic Lymphocytic Leukemia, Frontiers in Cell and Developmental Biology. (2025) 13, 1577081, 10.3389/fcell.2025.1577081, 40313718. PMC12043688 40313718

[52] Lim A. R. , Rathmell W. K. , and Rathmell J. C. , The Tumor Microenvironment as a Metabolic Barrier to Effector T Cells and Immunotherapy, Elife. (2020) 9, 10.7554/eLife.55185, 32367803. PMC7200151 32367803

[53] Wang C. D. , Zhang B. X. , and Song J. , Lipid Metabolic Reprogramming in Colorectal Cancer: Insights to Mechanisms and Therapeutics, World Journal of Gastrointestinal Oncology. (2025) 17, no. 10, 109398, 10.4251/wjgo.v17.i10.109398, 41114120. PMC12531765 41114120

[54] Zhuang Z. , Chen Y. , Yao Y. , and Zhu X. , Metabolic Reprogramming in Colorectal Cancer: The Impact of Fatty Acid Metabolism, Human Mutation. (2025) 2025, no. 1, 9567214, 10.1155/humu/9567214, 41031059. PMC12479164 41031059

[55] Medina Evora G. , Brauer M. , and Letellier E. , Host and Microbiome Lipid Metabolism in Colorectal Cancer Development and Therapy, Trends in Cancer. (2025) 11, no. 11, 1041–1057, 10.1016/j.trecan.2025.08.005. 40935766

[56] Liu W. , Dong S. , Hao F. , Gao Y. , and Wei Q. , Lipid Metabolic Reprogramming in Colorectal Cancer: Mechanisms and Therapeutic Strategies, Frontiers in Immunology. (2025) 16, 1603032, 10.3389/fimmu.2025.1603032, 40718481. PMC12289696 40718481

[57] Yang F. , Li Y. , Shang X. , Zhu Y. , Hou W. , Liu Y. , Hua Q. , and Sun Z. , Plin2 Promotes Colorectal Cancer Progression through Cd36-Mediated Epithelial-Mesenchymal Transition, Cell Death & Disease. (2025) 16, no. 1, 10.1038/s41419-025-07836-1, 40640171. PMC12246428 40640171

[58] Xie Z. , Niu L. , Zheng G. , du K. , Dai S. , Li R. , Dan H. , Duan L. , Wu H. , Ren G. , Dou X. , Feng F. , Zhang J. , and Zheng J. , Single-Cell Analysis Unveils Activation of Mast Cells in Colorectal Cancer Microenvironment, Cell & Bioscience. (2023) 13, no. 1, 10.1186/s13578-023-01144-x, 38031173. PMC10687892 38031173

[59] Shabani M. , Bayat A. A. , Jeddi-Tehrani M. , Rabbani H. , Hojjat-Farsangi M. , Ulivieri C. , Amirghofran Z. , Baldari C. T. , and Shokri F. , Ligation of Human Fc Receptor Like-2 by Monoclonal Antibodies Down-Regulates B-Cell Receptor-Mediated Signalling, Immunology. (2014) 143, no. 3, 341–353, 10.1111/imm.12311, 24797767. PMC4212948 24797767

[60] Martínez-Zamudio R. I. , Stefa A. , Nabuco Leva Ferreira Freitas J. A. , Vasilopoulos T. , Simpson M. , Doré G. , Roux P. F. , Galan M. A. , Chokshi R. J. , Bischof O. , and Herbig U. , Escape from Oncogene-Induced Senescence Is Controlled by Pou2f2 and Memorized by Chromatin Scars, Cell Genomics. (2023) 3, no. 4, 100293, 10.1016/j.xgen.2023.100293, 37082139. PMC10112333 37082139

[61] Seguella L. , Capuano R. , Pesce M. , Annunziata G. , Pesce M. , de Conno B. , Sarnelli G. , Aurino L. , and Esposito G. , S100b Protein Stimulates Proliferation and Angiogenic Mediators Release Through Rage/Pakt/Mtor Pathway in Human Colon Adenocarcinoma Caco-2 Cells, International Journal of Molecular Sciences. (2019) 20, no. 13, 10.3390/ijms20133240, 31266264. PMC6651655 31266264

